# 831. Hepatitis C Virus Micro-elimination Within a Human Immunodeficiency Virus Clinic: Challenges in the Home Stretch

**DOI:** 10.1093/ofid/ofab466.1027

**Published:** 2021-12-04

**Authors:** Jaklin Hanna, Jin S Suh, Humberto Jimenez

**Affiliations:** 1 Mount Sinai hospital, New York, New York; 2 St. Joseph’s University Medical Center, Ridgewood, New Jersey; 3 St Joseph’s University Medical Center, Paterson, New Jersey

## Abstract

**Background:**

Hepatitis c virus (HCV) eradication among persons with HIV (PWH) is alluring since DAAs efficacy is high regardless of HIV status and PWH in care are usually screened for HCV. Despite the potential, barriers to care have prevented many from achieving sustained virologic response (SVR). We performed a pharmacist-led campaign to reduce the proportion of PWH with active HCV and describe the barriers to care.

**Methods:**

This retrospective review evaluated patients receiving care at a Ryan White-funded clinic from 07/2018 to 12/2020. Patients were eligible if HCV diagnosed ≥1 year and receiving HIV care. The primary endpoint was to compare the prevalence HCV before and after a pharmacy initiative to target the remaining patients at the clinic not treated during first 3 ½ year period of oral DAA therapy availability. Secondary analysis was to identify barriers to care, measure the proportion of patients in each step of the HCV care cascade, and determine predictors of SVR. Among barriers to care, inconsistent engagement was defined as patients with habitual missed appointments. Logistic regression and Chi-square tests were performed.

**Results:**

46 of 1,100 PWH had active HCV for ≥1 year. Median age, years since HIV and HCV diagnoses were 58.5 years of age, 17 years, and 11.5 years, respectively. Most patients were male (70%), Black (61%), Latinx (28%), HCV genotype 1 (90%), had an HIV RNA < 200 copies/mL (72%), & had Medicaid (87%). 32/46 patients agreed to therapy, with all getting insurance approval and DAAs delivered. Glecaprevir/pibrentasvir (73%) was the preferred by payors, followed by sofosbuvir/velpatasvir (15%). Eight remained with active HCV and 19 achieved SVR. The prevalence rate dropped from 4.2% to 0.7% (P < 0.0001). Active drug use, inconsistent engagement, mental health disorder and nonadherence were initial barriers to care. After multivariate analysis, patients with inconsistent engagement continued to be less likely achieve SVR compared to those we remained consistently in care (aOR: 0.062, 95 CI: 0.009-0.421).

HCV care cascade in PWH within a Ryan White-funded clinic

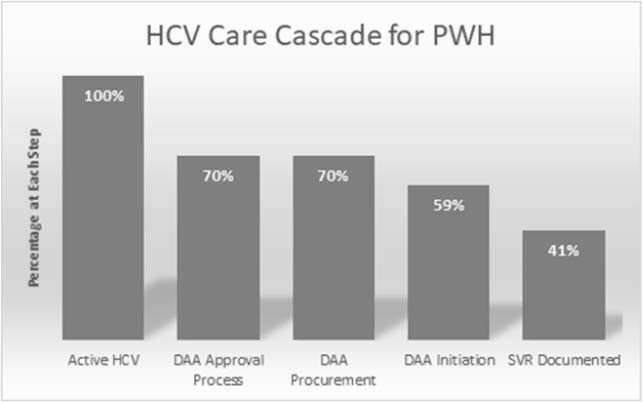

Active HCV includes 46 patients with chronic HCV infection receiving HIV in care at clinic, DAA approval process describes patients agreeing to HCV treatment along a continuum of pending laboratory results or pending prior authorization requests, DAA procurement depicts patients that have received approval and delivery of medications, DAA initiation describes patients who started treatment (27 patients), and SVR documented defines patients with an undetectable HCV RNA 12 weeks after therapy (19 patients).

**Conclusion:**

Pharmacists can impact the burden of HCV among PWH receiving care. The HCV care cascade remains tied to the HIV continuum of care, with disengagement from care remaining an important rate-limiting step impeding micro-elimination.

**Disclosures:**

**All Authors**: No reported disclosures

